# Pay-for-Performance Incentives for Home Dialysis Use and Kidney Transplant

**DOI:** 10.1001/jamahealthforum.2024.2055

**Published:** 2024-06-30

**Authors:** Kalli G. Koukounas, Daeho Kim, Rachel E. Patzer, Adam S. Wilk, Yoojin Lee, Kelsey M. Drewry, Rajnish Mehrotra, Maricruz Rivera-Hernandez, David J. Meyers, Ankur D. Shah, Rebecca Thorsness, Christopher H. Schmid, Amal N. Trivedi

**Affiliations:** 1 Department of Health Services, Policy and Practice, Brown University School of Public Health, Providence, Rhode Island; 2 Providence VA Medical Center, Providence, Rhode Island; 3 Regenstrief Institute, Indianapolis, Indiana; 4 Division of Transplant Surgery, Department of Surgery, Indiana University School of Medicine, Indianapolis; 5 Department of Health Policy and Management, Rollins School of Public Health, Emory University, Atlanta, Georgia; 6 Kidney Research Institute, Division of Nephrology, Department of Medicine, University of Washington School of Medicine, Seattle; 7 Warren Alpert Medical School of Brown University, Providence, Rhode Island; 8 Division of Kidney Disease and Hypertension, Rhode Island Hospital, Providence; 9 Partnered Evidence-Based Policy Resource Center, VA Boston Healthcare System, Boston, Massachusetts; 10 Department of Biostatistics, Brown University School of Public Health, Providence, Rhode Island

## Abstract

**Importance:**

The Centers for Medicare & Medicaid Services’ mandatory End-Stage Renal Disease Treatment Choices (ETC) model, launched on January 1, 2021, randomly assigned approximately 30% of US dialysis facilities and managing clinicians to financial incentives to increase the use of home dialysis and kidney transplant.

**Objective:**

To assess the ETC’s association with use of home dialysis and kidney transplant during the model’s first 2 years and examine changes in these outcomes by race, ethnicity, and socioeconomic status.

**Design, Setting, and Participants:**

This retrospective cross-sectional study used claims and enrollment data for traditional Medicare beneficiaries with kidney failure from 2017 to 2022 linked to same-period transplant data from the United Network for Organ Sharing. The study data span 4 years (2017-2020) before the implementation of the ETC model on January 1, 2021, and 2 years (2021-2022) following the model’s implementation.

**Exposure:**

Receiving dialysis treatment in a region randomly assigned to the ETC model.

**Main Outcomes and Measures:**

Primary outcomes were use of home dialysis and kidney transplant. A difference-in-differences (DiD) approach was used to estimate changes in outcomes among patients treated in regions randomly selected for ETC participation compared with concurrent changes among patients treated in control regions.

**Results:**

The study population included 724 406 persons with kidney failure (mean [IQR] age, 62.2 [53-72] years; 42.5% female). The proportion of patients receiving home dialysis increased from 12.1% to 14.3% in ETC regions and from 12.9% to 15.1% in control regions, yielding an adjusted DiD estimate of −0.2 percentage points (pp; 95% CI, −0.7 to 0.3 pp). Similar analysis for transplant yielded an adjusted DiD estimate of 0.02 pp (95% CI, −0.01 to 0.04 pp). When further stratified by sociodemographic measures, including age, sex, race and ethnicity, dual Medicare and Medicaid enrollment, and poverty quartile, there was not a statistically significant difference in home dialysis use across joint strata of characteristics and ETC participation.

**Conclusions and Relevance:**

In this cross-sectional study, the first 2 years of the ETC model were not associated with increased use of home dialysis or kidney transplant, nor changes in racial, ethnic, and socioeconomic disparities in these outcomes.

## Key Points

**Question:**

How was the Centers for Medicare & Medicaid Services’ End-Stage Renal Disease Treatment Choices (ETC) model associated with use of home dialysis and kidney transplant in the first 2 years of implementation?

**Findings:**

In this cross-sectional study of 724 406 traditional Medicare beneficiaries with kidney failure, there were no statistically significant differences in the use of home dialysis or kidney transplant between those treated in regions randomly assigned to the ETC model and those treated in control regions.

**Meaning:**

In its first 2 years of implementation, the ETC payment model has shown little impact in further promoting the uptake of home dialysis and kidney transplant among patients with kidney failure.

## Teaser

This cross-sectional study assesses the association of the Centers for Medicare & Medicaid Services’ End-Stage Renal Disease Treatment Choices model with use of home dialysis and kidney transplant during the model’s first 2 years and examines changes in these outcomes by race, ethnicity, and socioeconomic status.

## Introduction

Kidney failure (also known as end-stage kidney disease) is a lethal condition in which dialysis treatment or kidney transplant is required to sustain life. In 2021, more than 800 000 individuals in the US lived with kidney failure.^1^ Most patients are treated with in-center hemodialysis, a time-intensive modality requiring thrice-weekly, in-person treatments at dialysis centers followed by prolonged postdialysis symptoms.^2^ Research has suggested that some patients and clinicians prefer dialyzing at home,^3,4,5^ which allows for greater flexibility and independence, shorter recovery times, and some improved mortality outcomes.^6,7^ As of 2021, about 14.1% of prevalent patients received home dialysis,^1^ with higher use among White patients compared to Black or Hispanic patients, even though as many as 85% of patients are thought to be medically eligible for this service.^8,9^ Furthermore, the national prevalence of kidney transplant among patients undergoing dialysis was 4.0 per 100 person-years in 2021, below that of other high-income countries.^1^

In 2021, the Centers for Medicare & Medicaid Services (CMS) launched the End-Stage Renal Disease Treatment Choices (ETC) model, which sought to increase the use of home dialysis, kidney transplant, and transplant wait-listing among traditional Medicare beneficiaries.^4^ The model randomly selected 30% of the nation’s hospital referral regions (HRRs) for mandatory participation, such that all dialysis facilities and managing clinicians in selected HRRs are evaluated on model performance measures. Participants received payment incentives and penalties on the basis of their attributed patients’ use of home dialysis and kidney transplant or wait-listing.^10^ The model will run from 2021 though 2027, during which time outcomes in all participating facilities will be measured against their own historic performance and the performance of nonparticipating peers. Achievement and improvement relative to these benchmarks are scored and translated into payment adjustments, ranging from −5% to 4% in the first 2 years of the model.^10^

Evaluations of the model’s first year have reported mixed findings. One study found a small increase in initiation of home dialysis among incident patients in ETC relative to control regions.^11^ Another study, examining incident Medicare beneficiaries 66 years and older, found no meaningful difference in home dialysis use stemming from the ETC model.^12^ Our previous research evaluating ETC’s impact on participating safety-net facilities (those serving a high proportion of patients with social risk factors) found that the model disproportionately penalized high-risk facilities.^13^ Published evaluations to date have focused on first-year model outcomes, have been limited to the incident patient population, and did not separately examine effects for racial and ethnic minoritized populations or those with low income.

In this cross-sectional study, we evaluate the association of the ETC model with the use of home dialysis and kidney transplant during the model’s first 2 years. Given extensive evidence of racial and socioeconomic disparities in access to home dialysis and transplant,^3,8,14^ we additionally examined changes in these outcomes for Black, Hispanic, and Medicare and Medicaid dual eligible beneficiaries.

## Methods

### Study Design and Policy Timeline

This study analyzed claims and enrollment data for traditional Medicare beneficiaries from 2017 to 2022, linked to same-period transplant data from the United Network for Organ Sharing. The United Network for Organ Sharing serves as the nation’s transplant system, collecting and reporting data on every organ transplant conducted in the US.^15^ The study data span 4 years (2017-2020) before the implementation of the ETC model on January 1, 2021, and 2 years (2021-2022) following the model’s implementation. Additional detail on study methods can be found in the eMethods in Supplement 1.

The ETC model was proposed in July 2019 and finalized in September 2020 with the randomized selection of participating HRRs, stratified by census region.^16^ Despite randomization, there were preintervention imbalances in facility and patient characteristics, as well as use of home dialysis between ETC and non-ETC regions.^17^ To account for this, we used a difference-in-differences (DiD) approach to estimate changes in outcomes among patients treated in regions randomly selected for ETC participation, compared with concurrent changes among patients treated in control regions. The model was implemented on January 1, 2021, and is set to run through 2027 (eFigure 1 in Supplement 1), during which time CMS will monitor yearly performance for participating facilities and make corresponding payment adjustments to all reimbursements under Medicare’s End-Stage Renal Disease Prospective Payment System. In the first 2 years, these adjustments ranged from bonuses of 4% to penalties of −5%, with this range widening as the model progresses.^10^ The institutional review board at Brown University approved the study.

### Study Population and Exposure

The sample was constructed at the patient-month level, consisting of all qualifying months associated with individuals who met ETC model inclusion criteria, in an approach that mirrors how CMS conducts model measurement and attribution. We began by identifying the set of traditional Medicare beneficiaries with an end-stage kidney disease enrollment designation and aggregated their associated data at the month level for all months with 1 or more claims demonstrating the receipt of maintenance dialysis during the study period.^18,19^ Consistent with ETC model criteria, we excluded any person-months where beneficiaries did not have both Parts A and B coverage, were enrolled in Medicare Advantage (MA), lived outside of the US, were younger than 18 years, received dialysis care in hospice or nursing home settings, or had acute kidney injury, Alzheimer disease, or dementia (eFigure 2 in Supplement 1).^10,16^ Exclusions were done at the person-month level, such that individuals meeting the criteria previously listed may not have been fully excluded from the sample if for some months they were not subject to an exclusion criteria (eg, they began dialysis with no Alzheimer disease or dementia diagnosis but later received one). We used the HRR of the patient’s dialysis center to track ETC participation status, using CMS’s published list of 95 randomly selected HRRs to define the study’s treatment group, with all others as the control group.^20^ For patients who saw multiple dialysis centers in a single month, we prioritized the HRR of their most-used dialysis center in that month, excluding 8221 person-months (757 individuals) for whom an associated HRR could not be found. Transition from ETC-participating (ETC) to non–ETC-participating (control) regions was not common in the study sample, as fewer than 5% of all study participants experienced any transition over the 5-year study period. The final study sample included 724 406 unique patients.

### Outcome Measures

Study measures compared outcomes in ETC regions to those in control regions across the prepolicy (2017-2020) and postpolicy (2021-2022) periods. Primary outcomes of interest were home dialysis and kidney transplant, reflecting those studied by CMS in the ETC model. While CMS measures kidney transplant and transplant wait-listing as part of its ETC model evaluation, data limitations prevented us from incorporating transplant wait-listing in this study. Secondary outcomes included measures that could plausibly be affected by the ETC model, including 3-month mortality, hospital admissions, and disenrollment to MA (eTable 1 in Supplement 1). Three-month mortality was calculated excluding the last 3 months of 2022 to account for 2023 data censoring. The concurrent launch of the 21st Century Cures Act on January 1, 2021, which expanded MA enrollment to persons with kidney failure and resulted in a large exit of these patients to MA, prompted interest in the evaluation of any differential disenrollment to MA across ETC and non-ETC regions.^21^ All outcome measures are calculated at the month level, such that they reflect the unique number of monthly occurrences divided by the total person-months for the study group, except 3-month mortality, which is calculated using the 3-month period from the month of observation.

### Statistical Analysis

We conducted a DiD analysis comparing changes in monthly outcome measures between ETC and control regions (first difference) across prepolicy and postpolicy periods (second difference) to account for baseline differences across ETC and control regions.^17^ We adjusted the analysis for monthly age, sex, race and ethnicity (derived from the Centers for Medicare & Medicaid Services’ member enrollment file), dual Medicare and Medicaid enrollment (hereafter, dual status), reason for Medicare entitlement, zip code–level poverty and college completion (obtained from the American Community Survey),^22^ and monthly county-level COVID-19 mortality rates (obtained from *The New York Times*)^23^ (eTable 2 in Supplement 1). We additionally included month and census-region fixed effects to reflect the cyclical timing of dialysis treatment and the level at which model randomization was stratified. Standard errors were clustered at the level of treatment randomization (HRR) (eAppendix in Supplement 1).

To assess disparities in outcomes across measures of social risk, triple-difference analyses for home dialysis use were conducted across demographic and socioeconomic strata. These stratifications included age (<65 years vs ≥65 years), sex, race and ethnicity (Hispanic, non-Hispanic Black, and non-Hispanic White), dual status (nondual, any dual, partial dual, and full dual), and poverty quartile. The same covariates and fixed effects from the main DiD analysis were included, exempting those that overlapped with the strata of interest (eAppendix in Supplement 1).

Additional sensitivity analyses included (1) assessments of the preimplementation parallel trends between ETC and control regions^24^; (2) a change in the end point of the prepolicy period to reflect model announcement (July 2019) or (3) model finalization through HRR randomization (September 2020); (4) sample restriction to incident patients undergoing dialysis, studying outcomes for the 3-month period following dialysis initiation; (5) evaluation of 1-year, pre-ETC and post-ETC use trends among patients who were not included in the ETC model due to transition to MA on January 1, 2021; and (6) the use of HRR fixed effects in place of census-region fixed effects. All analyses were conducted in Stata, version 17 (StataCorp), with a 2-sided *P* < .05 considered statistically significant.

## Results

### Study Population

The study population included 724 406 persons with kidney failure (mean [IQR] age, 62.2 [53-72] years; 42.5% female), reflecting 18 118 190 person-months from January 1, 2017, to December 31, 2022. Across the full study period, ETC regions, compared with control regions, had a higher proportion of non-Hispanic Black patients (35.4% vs 30.5%) and a lower proportion of non-Hispanic White patients (48.7% vs 50.4%) and Hispanic patients (5.9% vs 8.6%); the ETC sample overrepresented the Southern (47.8% vs 43.8%) and Northern (17.2% vs 14.7%) US Census Bureau regions compared to the Midwest (17.8% vs 20.8%) and West (17.2% vs 20.6%). Finally, ETC regions vs control regions had slightly fewer dual beneficiaries (41.5% vs 42.8%), primarily driven by fewer full dual beneficiaries (32.7% vs 35.1%). Other covariates demonstrated relative balance across cohorts (Table 1). These trends remained constant across both postpolicy (eTable 3 in Supplement 1) and prepolicy (eTable 4 in Supplement 1) time periods.

**Table 1.** Characteristics of Traditional Medicare Beneficiaries With Kidney Failure Treated in End-Stage Renal Disease Treatment Choices (ETC) Model and Non-ETC Regions, 2017-2022 (N = 724 406)

Characteristic	No. (%)	
	Non-ETC	ETC
Total No. of individuals	483 799 (66.8)	240 607 (33.2)
Age, mean (SD), y	62.3 (14.4)	62.1 (14.4)
Sex		
Female	203 397 (42.0)	101 699 (42.3)
Male	280 402 (58.0)	138 908 (57.7)
Race and ethnicity^a^		
Asian	20 945 (4.3)	7455 (3.1)
Black, non-Hispanic	147 745 (30.5)	85 158 (35.4)
Hispanic	41 597 (8.6)	14 289 (5.9)
Native American	5433 (1.1)	5791 (2.4)
White, non-Hispanic	243 600 (50.4)	117 081 (48.7)
Other	13 241 (2.7)	5199 (2.2)
Unknown	11 238 (2.3)	5634 (2.3)
Census region		
Midwest	100 748 (20.8)	42 932 (17.8)
North	71 220 (14.7)	41 475 (17.2)
South	212 071 (43.8)	114 908 (47.8)
West	99 760 (20.6)	41 292 (17.2)
Medicare entitlement reason(s)		
End-stage kidney disease	465 001 (96.1)	232 254 (96.5)
Age	233 091 (48.2)	115 353 (47.9)
Disability	146 709 (30.3)	73 745 (30.6)
Social determinants of health factors		
Zip code–level poverty^b^	18.0 (9.8)	18.1 (10.1)
Zip code–level college completion^c^	22.7 (13.4)	22.6 (12.5)
Any Medicare and Medicaid dual enrollment	206 895 (42.8)	99 952 (41.5)
Partial Medicare and Medicaid dual enrollment	37 038 (7.7)	21 366 (8.9)
Full Medicare and Medicaid dual enrollment	169 857 (35.1)	78 586 (32.7)

^a^ Race and ethnicity designations are derived from the Centers for Medicare & Medicaid Services’ member enrollment file associated with Medicare claims data for 2017 through 2022.

^b^ Data are derived from the American Community Survey and represent the percentage of the zip code’s population at or below 100% of the federal poverty level.

^c^ Data are derived from the American Community Survey and represent the percentage of the zip code’s population who have completed at least a bachelor’s degree.

### Outcomes

**Figure 1.**
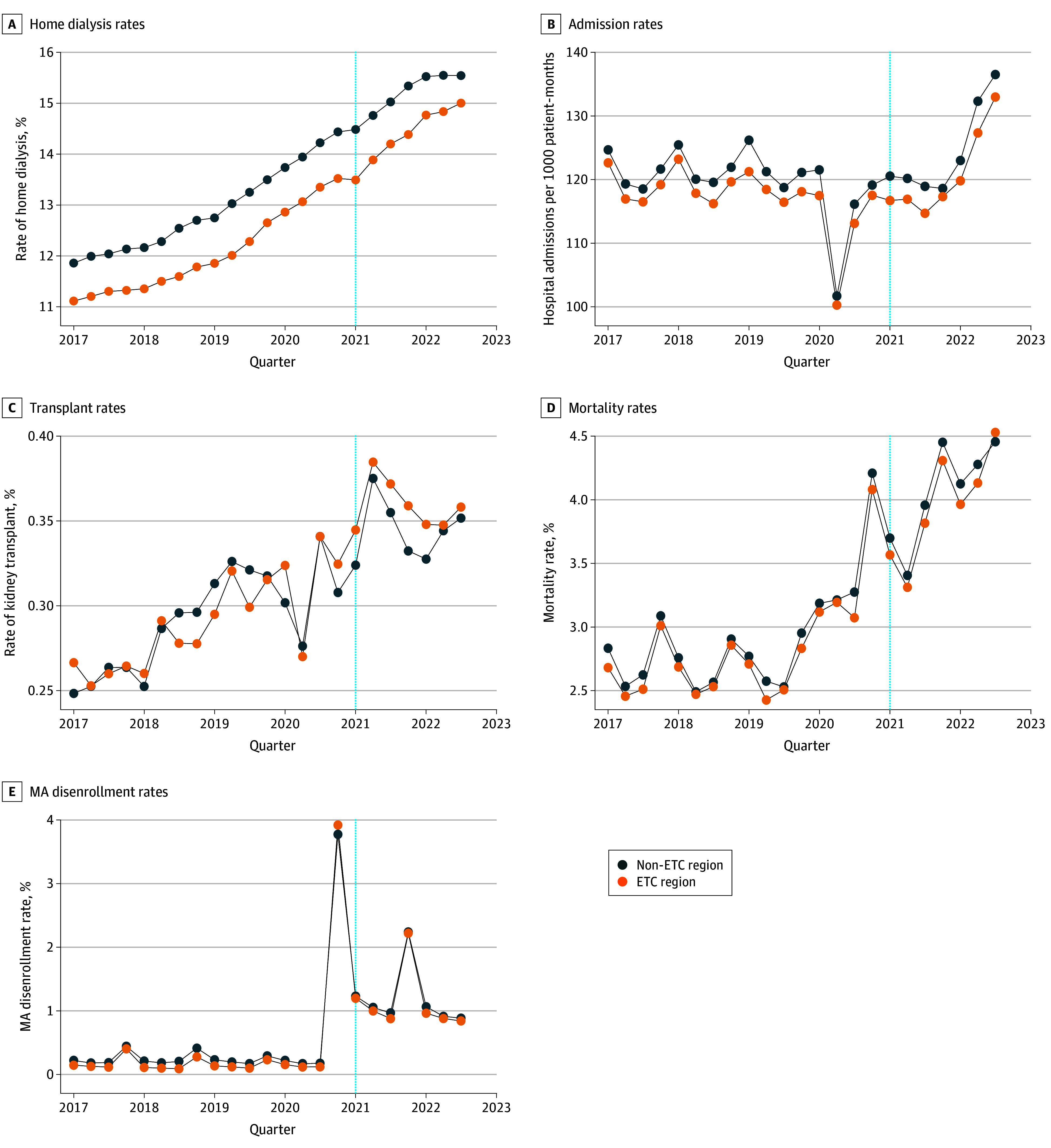
Unadjusted Outcome Trends by End-Stage Renal Disease Treatment Choices (ETC) Model Region Over the Study Period by Quarter, 2017-2022 The graphs exclude the fourth quarter of 2022 (October, November, and December) to address data missingness and censoring. MA indicates Medicare Advantage. The blue dashed line in all panels represents the launch of the ETC.

Unadjusted trends over time demonstrated that control regions had consistently higher baseline use of home dialysis than ETC regions—on average 12.9% compared to 12.1% in the prepolicy period (Figure 1). The test of parallel preimplementation outcome trends in eFigure 3 in Supplement 1 did not suggest a statistically significant change in this gap over time between ETC and control regions.

There were no statistically significant associations between implementation of the ETC model and changes in any of the primary and secondary outcomes (Table 2). Between the prepolicy and postpolicy periods, home dialysis use increased by 2.2 percentage points (pp), from 12.1% to 14.3%, in ETC regions and 2.2 pp, from 12.9% to 15.1%, in control regions, resulting in an adjusted DiD estimate of −0.2 pp (95% CI, −0.7 to 0.3 pp). For kidney transplant, there was an increase of 0.03 pp in ETC regions (0.29% to 0.32%) and 0.01 pp in control regions (0.29% to 0.30%), resulting in an adjusted DiD estimate of 0.02 pp (95% CI, −0.01 to 0.04 pp). Unadjusted trends over time for primary and secondary outcomes can be seen in Figure 1.

**Table 2.** Difference-in-Differences Results Among Traditional Medicare Beneficiaries With Kidney Failure Treated in End-Stage Renal Disease Treatment Choices (ETC) Model and Control Regions, 2017-2022

Variable	Regions, %						Difference-in-difference (95% CI)	
	ETC			Control				
	Prepolicy	Postpolicy	Difference, pp	Prepolicy	Postpolicy	Difference, pp	Unadjusted	Adjusted
No. of person-months	4 425 700	1 702 832	NA	8 682 187	3 307 471	NA	NA	NA
Home dialysis	12.1	14.3	2.2	12.9	15.1	2.2	0.07 (−0.41 to 0.56)	−0.20 (−0.73 to 0.33)
Kidney transplant	0.29	0.32	0.03	0.29	0.30	0.01	0.01 (−0.01 to 0.04)	0.02 (−0.01 to 0.04)
3-mo Mortality^a^	2.8	3.9	1.1	2.9	4.0	1.1	−0.03 (−0.13 to 0.08)	−0.04 (−0.13 to 0.05)
Hospital admissions (per 1000 patient-months)	117.2	121.3	4.2	119.9	125.0	5.1	−1.24 (−3.77 to 1.30)	0.22 (−2.55 to 2.99)
Monthly disenrollment to MA, %	0.4	1.0	0.6	0.5	1.1	0.6	0.01 (−0.10 to 0.13)	0.003 (−0.11 to 0.11)

Abbreviations: MA, Medicare Advantage; NA, not applicable; pp, percentage point.

^a^ For the 3-month mortality outcome, data from the last 3 months of 2022 (October, November, and December) were removed to account for data censoring, given the lack of 2023 data on date of death, and thus likely undercount the 3-month rate of death in these last 3 months.

**Figure 2.**
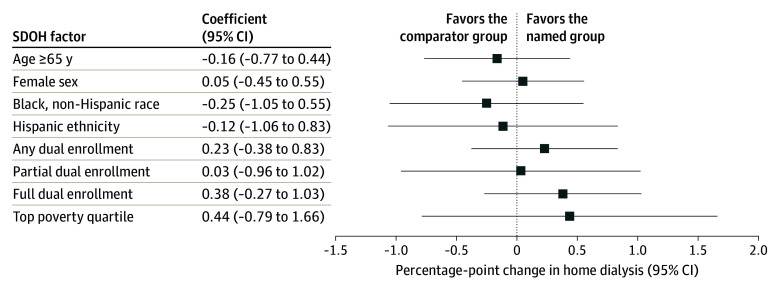
Triple Difference-in-Differences Home Dialysis Results Among Patients With Kidney Failure Stratified by Social Determinants of Health (SDOH) Factors, 2017-2022 Each coefficient presented represents a comparison of home dialysis use in the End-Stage Renal Disease Treatment Choices (ETC) model in ETC regions relative to another cohort of interest. Age 65 years and older is compared to age younger than 65 years; female sex is compared to male sex; Black race and Hispanic ethnicity are compared to White race; any dual, partial dual, and full dual enrollment are compared to nondual enrollment; and the top poverty quartile is compared to all other quartiles.

Figure 2 demonstrates the results of triple-difference analyses on home dialysis use across different stratifications of social risk. No statistically significant associations with the ETC model were observed for any of the subgroups. Expanded results are summarized in eTable 5 in Supplement 1. Unadjusted home dialysis trends over time for stratified populations can be seen in eFigure 4 in Supplement 1.

### Sensitivity Analyses

When comparing patients with kidney failure who transitioned to MA on January 1, 2021, to those who remained in traditional Medicare, prepolicy differences were observed in both patient characteristics (eTable 6 in Supplement 1) as well as use of home dialysis, kidney transplant, and hospital admission (eTable 7 in Supplement 1). Differences in patient characteristics mirrored those shown in prior work, with higher proportions of patients who were non-Hispanic Black, were from the South, had a disability, and had dual eligibility or lower income transitioning to MA.^21^ In both ETC- and non-ETC–participating regions, home dialysis use in 2020 was 2.7 pp to 3.1 pp higher among patients who stayed in traditional Medicare compared to those who disenrolled to MA. Furthermore, in the year following MA transition, lower rates of mortality and kidney transplant were observed among those disenrolled to MA compared to those who remained in traditional Medicare (eTable 8 in Supplement 1).

When the population was limited to incident patients in the 3-month period following dialysis initiation, a small but statistically significant effect in favor of the ETC policy change was observed in the unadjusted home dialysis DiD among this cohort (1.1 pp; 95% CI, 0.02-2.2 pp), as well as in adjusted mortality (−0.3 pp; 95% CI, −0.6 to −0.03 pp) and hospital admissions (−7.0 pp; 95% CI, −13.0 to −0.9 pp). A description of the incident patient cohort can be found in eTable 9 in Supplement 1, and full DiD results are summarized in eTable 10 in Supplement 1. The main study results are robust to an adjusted prepolicy end date of either model announcement in July 2019 (eTable 11 in Supplement 1) or HRR randomization in September 2020 (eTable 12 in Supplement 1), as well as to adjustment of fixed effects from Census region to HRR (eTable 13 in Supplement 1).

## Discussion

In the first 2 years of CMS’s ETC model, we observed no statistically significant differences in the use of home dialysis or kidney transplant among model participants compared to nonparticipants. Despite temporal increases in home dialysis and kidney transplant use nationally, the ETC model was not associated with differentially increased use of these services among patients treated by model participants relative to those treated by dialysis facilities in control regions. Secondary outcomes, including 3-month mortality, hospital admissions, and disenrollment to MA, similarly showed no statistically significant differences between patients treated in ETC vs control regions. Finally, when further stratified by sociodemographic measures including age, sex, race and ethnicity, dual status, and poverty quartile, home dialysis use did not meaningfully differ across joint strata of characteristics and ETC participation.

The concurrent launch of the 21st Century Cures Act on January 1, 2021, meant that a substantial portion of patients with kidney failure left traditional Medicare for MA at the exact time that the ETC model launched, making them ineligible for model participation.^21^ Lower prepolicy use of home dialysis and kidney transplant among the population that transitioned to MA suggests that some of the observed increases in these outcomes among traditional Medicare beneficiaries may be an artifact of individuals with lower service use disproportionately switching to MA after January 1, 2021.

This research expands on prior ETC evaluations in important ways. First, it includes both the first and second years of the model implementation and demonstrates that additional time and model experience has not improved outcomes relative to control regions. Second, it evaluates results across the entire traditional Medicare kidney failure population (incident and prevalent patients) using claims data, which allow us to track documented dialysis modality over time. Prior research has found modest effects of the ETC model in the first year when limited to incident patients but only when using data from CMS’s Medical Evidence Report (form 2728).^11^ This form reflects data collected at dialysis initiations and is not updated as treatment continues. Thus, the present research adds to the literature by studying the real-time modality patients receive over the model period. Finally, we evaluated differential policy impact by race and ethnicity and socioeconomic status.

The ETC model’s limited influence on home dialysis and kidney transplant use in the first 2 years may be attributed to several factors. First, home dialysis use in the US has been steadily increasing since 2009, likely triggered by that year’s expansion of the Prospective Payment System.^1,25^ As such, there is an existing financial incentive to increase home dialysis use that extends to all national dialysis facilities. Second, the use of home dialysis requires stable housing, as well as the ability to learn and self-administer complex medical regimens, family and caregiver support, and the financial resources for potential home modifications and higher utility bills.^26,27^ The application of pay-for-performance incentives to dialysis facilities does not address these patient-level barriers. Third, the process of kidney transplant is lengthy, complex, and requires several steps from referral to evaluation, wait-listing, and ultimately transplant. As such, it may take several years to observe any impact from the ETC model on access to transplant. Fourth, the consolidation of the dialysis treatment market means that 2 large parent companies operate approximately 70% of the facilities present in both ETC and control regions. Therefore, the payment penalties in one set of facilities may be offset by revenue or bonuses from other facilities owned by the same company. Additionally, it is possible that care-management practices may be standardized across all facilities operated by a large dialysis organization, which may reduce the possibility that these organizations introduced interventions in only their ETC-exposed facilities. These factors may potentially blunt the effect of financial incentives.

The ETC model is currently set to run through 2027, during which time the payment bonuses and penalties will ultimately escalate to 8% and −10%, respectively.^10^ The disproportionate penalization of facilities serving patients with high social risk^13^ and the lack of consistent evidence that the ETC model is improving outcomes raise concerns about the continued implementation of the model as is through its scheduled end date. CMS could consider providing additional resources to facilities, particularly those that treat patients with higher social risk, to help overcome structural barriers that prevent uptake of home dialysis or kidney transplant.^27,28,29^ Research should continue to monitor the ETC’s impact on patients and safety-net dialysis facilities to ensure that escalating penalizes do not contribute to widening disparities.

### Limitations

This study has several limitations. First, we cannot observe the potential ETC model spillovers to those not enrolled in traditional Medicare. Second, we are unable to track transplant wait-listing in the data and thus have not studied the ETC model’s effect on this precursor to kidney transplant receipt. Third, the study was limited to the first 2 years of the ETC model.

## Conclusions

In this cross-sectional study of 724 406 traditional Medicare beneficiaries with kidney failure, we observed no statistically significant changes in the use of home dialysis or kidney transplant in the first 2 years of CMS’s ETC model. These findings, coupled with prior research that demonstrates the disproportionate penalization of safety-net dialysis facilities, supports careful consideration of the model’s future implementation and efficacy within this at-risk population.

## Notes

Supplement 1.
eMethods
**eFigure 1.** Visual Representation of the Timeline for CMS’ ETC Model (2019 – 2027) **eFigure 2.** Sample Cohort Construction **eTable 1.** Outcome measure technical definitions **eTable 2.** Covariate measure technical definitions
eAppendix
**eTable 3.** Individual-Level Distribution of Characteristics Across ETC and Non-ETC Assigned Regions, Active-Model Time Period (2021-2022) **eTable 4.** Individual-Level Distribution of Characteristics Across ETC and Non-ETC Assigned Regions, Pre-ETC Model Time Period (2017 - 2020) **eFigure 3.** Event Study Plots **eTable 5.** Triple Difference-in-Difference Results for Home Dialysis Utilization (%) Among Kidney Failure Patients, Stratified by Social Determinants of Health Factors (2017-2022) **eFigure 4.** Unadjusted Home Dialysis Trends by Quarter, Stratified Analyses **eTable 6.** Individual-Level Distribution of Characteristics Across ETC and Non-ETC Assigned Regions, Distributed by Staying in Traditional Medicare vs Switching to Medicare Advantage in Jan. 2021 (2020) **eTable 7.** Pre-Period (2020) Person-Month Level Difference in Outcomes Between those who Stayed in Traditional Medicare vs those who Switched to Medicare Advantage in Jan. 2021, (2020) **eTable 8.** Post-Period (2021) Person-Month Level Difference in Outcomes Between those who Stayed in Traditional Medicare vs those who Switched to Medicare Advantage in Jan. 2021, (2021) **eTable 9.** Individual-Level Distribution of Characteristics Across ETC and Non-ETC Assigned Regions, Among Incident Kidney Failure Patients (2017-2022) **eTable 10.** Difference-in-Difference Results Among Incident Kidney Failure Patients, Months 1-3 Post Treatment Incidence (2017-2022) **eTable 11.** Difference-in-Difference Results Among Kidney Failure Patients, Sensitivity Analysis Using July 2019 Model Announcement Date (2017-2022) **eTable 12.** Difference-in-Difference Results Among Kidney Failure Patients, Sensitivity Analysis Using September 2020 HRR Randomization Announcement Date (2017-2022) **eTable 13.** Difference-in-Difference Results Among Kidney Failure Patients using HRR fixed effects (2017-2022)
eReferences

Supplement 2. Data Sharing Statement
