# Supplementary material for: Pay-for-Performance Incentives for Home Dialysis Use and Kidney Transplant
Source: JAMA Health Forum. 2024 Jun 30;5(6.9):e242055. doi: 10.1001/jamahealthforum.2024.2055 (PMC11215557; doi:10.1001/jamahealthforum.2024.2055)
Supplement: Supplement 1. — eMethods eFigure 1. Visual Representation of the Timeline for CMS’ ETC Model (2019 – 2027) eFigure 2. Sample Cohort Construction eTable 1. Outcome measure technical definitions eTable 2. Covariate measure technical definitions eAppendix eTable 3. Individual-Level Distribution of Characteristics Across ETC and Non-ETC Assigned Regions, Active-Model Time Period (2021-2022) eTable 4. Individual-Level Distribution of Characteristics Across ETC and Non-ETC Assigned Regions, Pre-ETC Model Time Period (2017 - 2020) eFigure 3. Event Study Plots eTable 5. Triple Difference-in-Difference Results for Home Dialysis Utilization (%) Among Kidney Failure Patients, Stratified by Social Determinants of Health Factors (2017-2022) eFigure 4. Unadjusted Home Dialysis Trends by Quarter, Stratified Analyses eTable 6. Individual-Level Distribution of Characteristics Across ETC and Non-ETC Assigned Regions, Distributed by Staying in Traditional Medicare vs Switching to Medicare Advantage in Jan. 2021 (2020) eTable 7. Pre-Period (2020) Person-Month Level Difference in Outcomes Between those who Stayed in Traditional Medicare vs those who Switched to Medicare Advantage in Jan. 2021, (2020) eTable 8. Post-Period (2021) Person-Month Level Difference in Outcomes Between those who Stayed in Traditional Medicare vs those who Switched to Medicare Advantage in Jan. 2021, (2021) eTable 9. Individual-Level Distribution of Characteristics Across ETC and Non-ETC Assigned Regions, Among Incident Kidney Failure Patients (2017-2022) eTable 10. Difference-in-Difference Results Among Incident Kidney Failure Patients, Months 1-3 Post Treatment Incidence (2017-2022) eTable 11. Difference-in-Difference Results Among Kidney Failure Patients, Sensitivity Analysis Using July 2019 Model Announcement Date (2017-2022) eTable 12. Difference-in-Difference Results Among Kidney Failure Patients, Sensitivity Analysis Using September 2020 HRR Randomization Announcement Date (2017-2022) eTable 13. Difference-in-Difference [file jamahealthforum-e242055-s001.pdf]

Supplemental Online Content

Koukounas KG, Kim D, Patzer RE, et al. Pay-for-performance incentives for home dialysis use and kidney transplant. *JAMA Health Forum*. 2024;5(6.9):e242055.
doi:10.1001/jamahealthforum.2024.2055

eMethods

eFigure 1. Visual Representation of the Timeline for CMS' ETC Model (2019 – 2027)

eFigure 2. Sample Cohort Construction

eTable 1. Outcome measure technical definitions

eTable 2. Covariate measure technical definitions

eAppendix

eTable 3. Individual-Level Distribution of Characteristics Across ETC and Non-ETC Assigned Regions, Active-Model Time Period (2021-2022)

eTable 4. Individual-Level Distribution of Characteristics Across ETC and Non-ETC Assigned Regions, Pre-ETC Model Time Period (2017 - 2020)

eFigure 3. Event Study Plots

eTable 5. Triple Difference-in-Difference Results for Home Dialysis Utilization (%) Among Kidney Failure Patients, Stratified by Social Determinants of Health Factors (2017-2022)

eFigure 4. Unadjusted Home Dialysis Trends by Quarter, Stratified Analyses

eTable 6. Individual-Level Distribution of Characteristics Across ETC and Non-ETC Assigned Regions, Distributed by Staying in Traditional Medicare vs Switching to Medicare Advantage in Jan. 2021 (2020)

eTable 7. Pre-Period (2020) Person-Month Level Difference in Outcomes Between those who Stayed in Traditional Medicare vs those who Switched to Medicare Advantage in Jan. 2021, (2020)

eTable 8. Post-Period (2021) Person-Month Level Difference in Outcomes Between those who Stayed in Traditional Medicare vs those who Switched to Medicare Advantage in Jan. 2021, (2021)

eTable 9. Individual-Level Distribution of Characteristics Across ETC and Non-ETC Assigned Regions, Among Incident Kidney Failure Patients (2017-2022)

eTable 10. Difference-in-Difference Results Among Incident Kidney Failure Patients, Months 1-3 Post Treatment Incidence (2017-2022)

© 2024 Koukounas KG et al. *JAMA Health Forum.*

eTable 11. Difference-in-Difference Results Among Kidney Failure Patients, Sensitivity Analysis Using July 2019 Model Announcement Date (2017-2022)

eTable 12. Difference-in-Difference Results Among Kidney Failure Patients, Sensitivity Analysis Using September 2020 HRR Randomization Announcement Date (2017-2022)

eTable 13. Difference-in-Difference Results Among Kidney Failure Patients using HRR fixed effects (2017-2022)

eReferences

This supplemental material has been provided by the authors to give readers additional information about their work.

eMethods

Model Timeline[1–3]

CMS proposed the ETC model on July 10th, 2019, as part of the Specialty Care Models to Improve Quality of Care and Reduce Expenditures rule. The model received public comments through Sept. 16th, 2019 and was published as part of Final Rule 85 61114 on Sept. 18th, 2020. The model went into effect on Jan. 1st, 2021. In July 2021, ETC model changes were proposed to address health equity concerns as part of the ESRD Prospective Payment System (PPS) Notice of Proposed Rulemaking. The changes received public comment through August 31st, 2021, and were finalized through the CY 2022 End-Stage Renal Disease Prospective Payment System Final Rule on Oct. 29th, 2021. These adjustments to the model's scoring and benchmarking methodology were implemented on Jan. 1st, 2022, such that the first year of the model did not utilize these changes.[1] The model is set to run between Jan. 1st, 2021, and June 30th, 2026, with payment periods corresponding to 6 months after each annual measurement period. Thus, payment periods for the ETC model will extend from July 1st, 2022, to June 30th, 2027.

The ETC model utilizes three different period types to classify time. The first is "Benchmark Year" (BY), which is used to construct the benchmark rates against which model participants will be compared. The second is "Measurement Year" (MY), which refers to the period during which the performance of ETC facilities is tracked and measured. Finally, "Performance Payment Adjustment Period" (PPA) refers to the period during which financial penalties and bonuses are awarded based on the facility's scoring and performance in the corresponding MY. BYs are 12-month periods that occur 18 months prior to the start of each MY, and PPAs are 6-month periods that occur 6 months after each MY. The 6 months between each BY, MY and PPA are used to allow for 3 months of claims runout and 3 months of calculation. Finally, model years overlap for 6 months of every year, such that after July 1st, 2021, there are two MYs running concurrently at any given time. This is done to ensure that the PPAs occur in succession without gaps. A visualization of the ETC model schedule, taken from CMS, is shown below.[3]

<u>eFigure 1: Visual Representation of the Timeline for CMS' ETC Model (2019 – 2027)[a]</u>

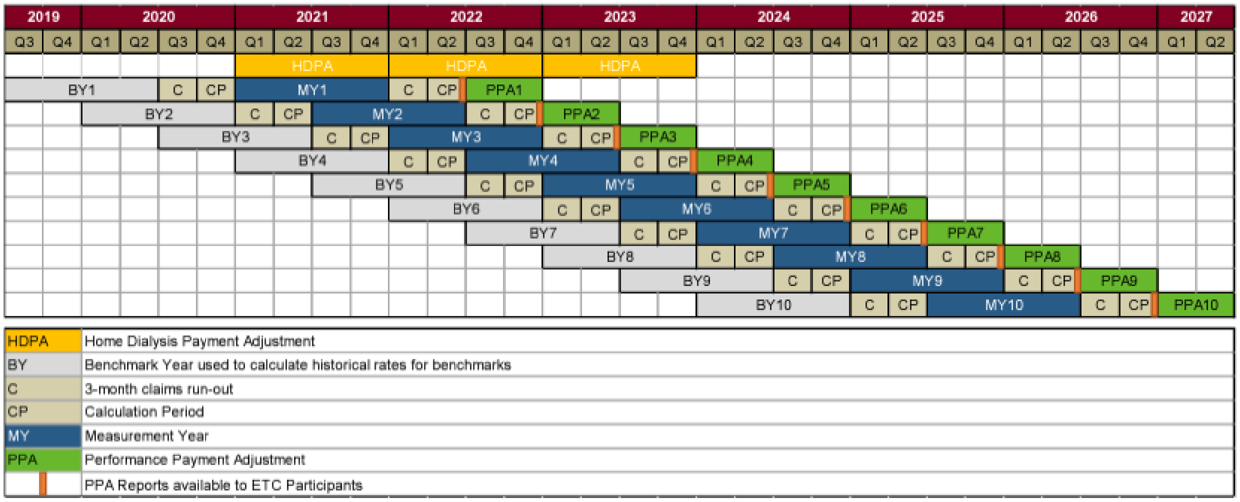

[a] Image obtained from CMS' End-Stage Renal Disease Treatment Choices (ETC) Model Performance Payment Adjustment Report User Guide (Measurement Years 1–2)

Sample Construction

© 2024 Koukounas KG et al. *JAMA Health Forum.*

We used claims and enrollment data for traditional Medicare beneficiaries enrolled between Jan. 1st, 2017 – Dec. 31st 2022. Using unique person identifiers, we linked to all available data from the United Network for Organ Sharing (UNOS) which included information on available kidney transplants occurring during the same time period. We first limited our sample to all traditional Medicare beneficiaries with an ESRD enrollment designation and evidence of the receipt of maintenance dialysis during the study period. We defined the receipt of maintenance dialysis as specified by CMS' federal register for ETC model announcement,[4] excluding acute kidney injury (AKI) claims as specified by the Medicare claims processing manual.[5] For this cohort, we developed a person-month level panel dataset for all months demonstrating evidence of the receipt of dialysis treatment. Employing the same exclusions that CMS has defined for the ETC model,[6] we then limited this dataset to all person-months where individuals were eligible for ETC model participation. Exclusions, shown in **eFigure 2** below, included individuals:

- (1) Lacking both Medicare Parts A & B
- (2) Receiving dialysis while enrolled in Medicare Advantage
- (3) Residing outside the U.S.
- (4) Under the age of 18
- (5) Receiving hospice care
- (6) Receiving dialysis for acute kidney injury (AKI)[5]
- (7) Diagnosed with Alzheimer's or Dementia
- (8) Receiving dialysis in a nursing home

The resultant cohort included 18,126,411 person-months. ETC vs non-ETC designation was done at the provider level, based on the hospital-referral region (HRR) of their most-used dialysis provider in that month. If ties existed, ETC-based providers were preferred, and otherwise the provider with the earliest claim date.

<u>eFigure 2. Sample Cohort Construction</u>

Person-Month Level

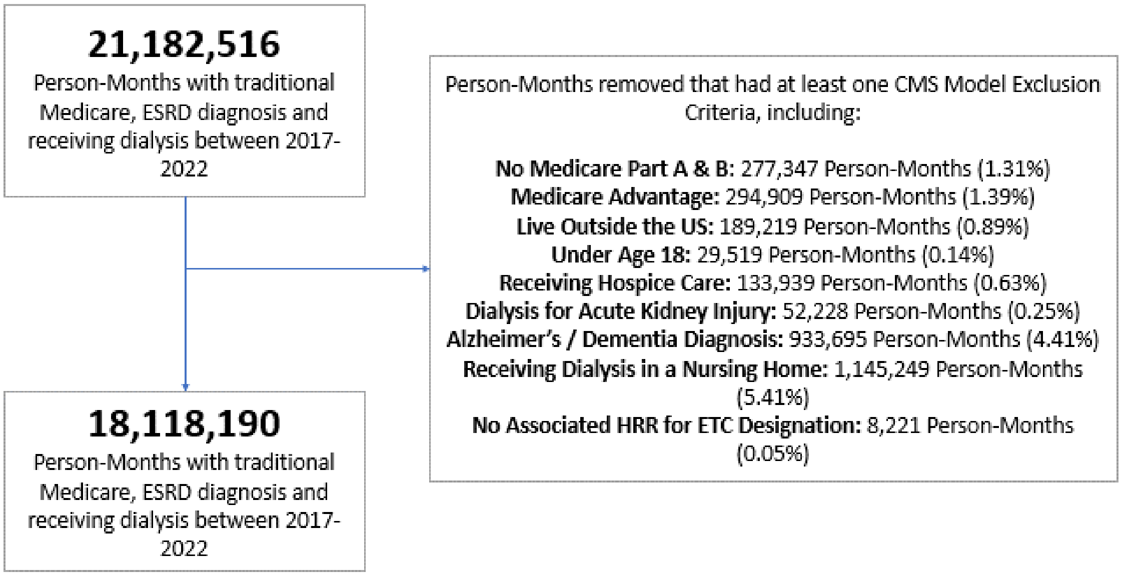

The data source utilized for the hospice exclusion only included patients through 2020, however given the small size of this exclusion population and the likelihood that they are accounted for in other exclusions, we were not concerned about this censoring. Further, the data source utilized for the Nursing Home

© 2024 Koukounas KG et al. *JAMA Health Forum.*

exclusion only included patients through Q3 2022, meaning we are likely slightly undercounting nursing home patients in our analysis during the last 3 months of 2022.

Unique Person Level

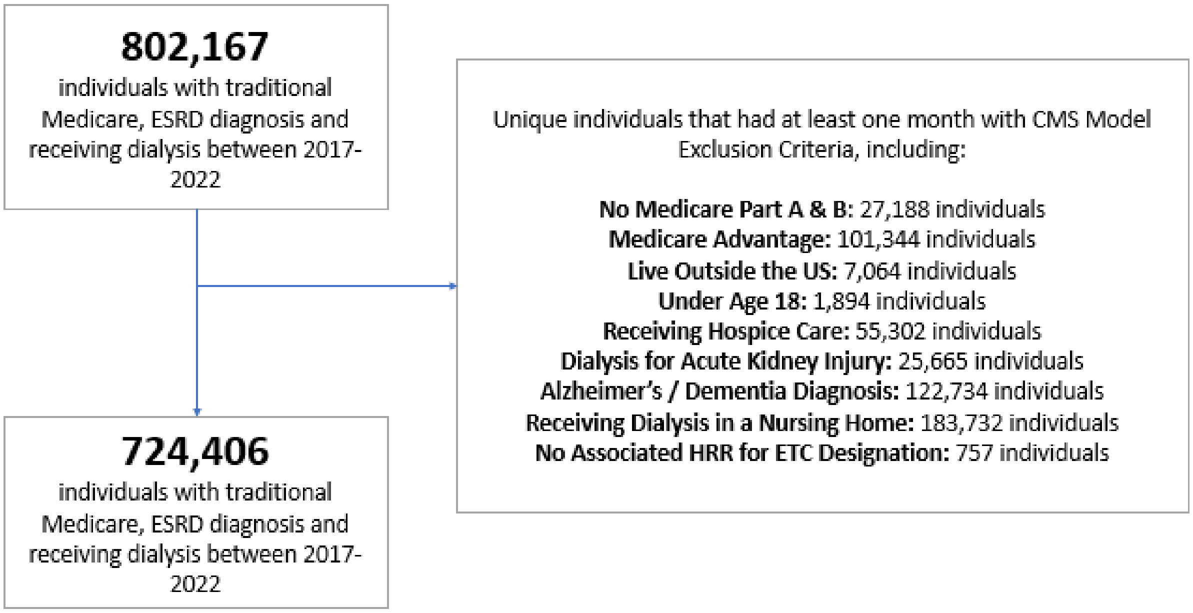

Exclusions were made at the person-month level, the same level of the dataset, such that if an individual had 12 months of traditional Medicare enrollment before switching to Medicare Advantage, those 12 months would be retained, while any person-months after Medicare Advantage enrollment were excluded. Thus, all persons listed in the person-level flow chart above were not necessarily excluded fully from the dataset, but may have had some portion of their monthly data excluded.

Outcomes Measures

Our study employed two primary and three secondary outcomes. The primary outcomes were use of home dialysis and kidney transplant, to align with those of CMS' ETC model. Secondary outcomes included measures that could be plausibly impacted by the ETC model, including 3-month mortality, hospital admissions, and disenrollment to Medicare Advantage (MA). Outcome measures are defined in more detail in **eTable 1** below:

eTable 1. Outcome measure technical definitions

Outcome	Definition	Data Source
Home Dialysis	Identified in the claims data using part B claims with procedure codes 90957-90962, 90965 and 90966, or outpatient institutional claims with bill type 072X and condition codes 74 or 76, we flagged person-months where we observed the use of any home dialysis during the month period. Proportion of months with any home dialysis use were then taken over total person-months in the cohort of interest to produce reported metrics.	Medicare Claims Data, 2017-2022

© 2024 Koukounas KG et al. *JAMA Health Forum.*

Kidney Transplant	Identified using linked UNOS data, transplants were flagged on the month the transplant was received. Proportion of months with any transplant were then taken over total person-months in the cohort of interest to produce reported metrics	UNOS data, 2017- 3Q 2022
3-month mortality	Date of death was identified from the Medicare enrollment file. All months in the 3 months prior to the month of death were flagged. Proportion of months in the 3-month period prior to death were taken over total person-months in the cohort of interest to produce reported metrics. Metric was calculated excluding the final 3 months of 2022 to account for data censoring due to lack of 2023 outcomes.	Medicare Beneficiary Summary File, 2017-2022
Hospital admissions	Total number of unique hospital admissions per person per month were identified based on admission date. The summed count of admissions for all person-months in the cohort was taken over the total person-month count.	Medicare Claims Data, 2017-2022
Disenrollment to Medicare Advantage (MA)	Enrollment in Medicare Advantage was identified from the Medicare Enrollment file. For each beneficiary, the month of MA enrollment after the last month of dialysis utilization in TM was flagged, as well as the last month of dialysis utilization in traditional Medicare before transitioning to MA. The proportion of months flagged as the last TM service month prior to MA transition were taken over total person-months in the cohort of interest to produce reported metrics	Medicare Claims and Medicare Beneficiary Summary File, 2017-2022

Covariate Measures

Our study employed eight covariates, used to adjust for time-varying differences in population makeup across census regions and dialysis months. Covariates included age, sex, race and ethnicity, dual Medicare/Medicaid enrollment, reason for Medicare entitlement, ZIP-code level poverty, college completion, and monthly COVID-19 death rates. Covariate measures are defined in more detail in **eTable 2** below:

eTable 2. Covariate measure technical definitions

Outcome	Definition	Data Source
Monthly Age	Identified based on the Medicare Beneficiary Summary File's date of birth for each individual, back calculated to represent the person's age per month of dialysis.	Medicare Beneficiary Summary File, 2017-2022
Sex	Identified based on the annual designation for each beneficiary from the Medicare Beneficiary Summary File.	Medicare Beneficiary Summary File, 2017-2022

Race/Ethnicity	Identified based on the annual designation for each beneficiary from the Medicare Beneficiary Summary File.	Medicare Beneficiary Summary File, 2017-2022
Medicare-Medicaid Dual Status	Identified based on the monthly designation for each beneficiary from that year's Medicare Beneficiary Summary File. Dual Medicare-Medicaid Status occurs across the following enrollment levels:<br><br>Full Duals (Dual status codes 02, 04, 08): represent individuals who are eligible for full Medicaid benefits, such that they are eligible for all Medicaid benefits a state provides, not just those afforded to Medicare through cost-sharing.<br><br>Partial Duals (Dual status codes 01, 03, 05, 06): represent individuals who are not eligible for full Medicaid benefits, such that they are eligible for Medicaid only as a provider of coinsurance coverage for Medicare, which can include just Part A or Part B coverage, or both depending on poverty level.	Medicare Beneficiary Summary File, 2017-2022
Reason for Medicare Entitlement	Identified based on the monthly designation for each beneficiary from that year's Medicare Enrollment File. Medicare entitlement reasons include Aged, Disabled or ESRD, as well as combinations of these conditions. Members can have multiple reasons for Medicare entitlement, for instance both Age and ESRD. In the difference-in-differences analyses, we specifically adjusted for enrollment due to age and enrollment due to disability, as these represented distinct populations than those who were enrolled solely due to ESRD.	Medicare Beneficiary Summary File, 2017-2022
ZIP-Code Level Poverty	Identified from the American Community Survey at the ZCTA level, and then cross-referenced to the ZIP-code of residence associated with each individual from that year's Medicare Enrollment File. Poverty was represented by the percentage of individuals living in the ZIP Code with income less than or equal to 100% of the federal poverty limit.<br><br>For the difference-in-difference analyses, ZIP Code-level poverty was re-coded into quartiles. At the ZIP Code level, 7,340 person-months (0.04%) were missing an associated poverty rate. These were excluded from the analyses.	American Community Survey, 2015
ZIP-Code Level College Completion	Identified from the American Community Survey at the ZCTA level, and then cross-referenced to the ZIP-code of residence associated with each individual from that year's Medicare Enrollment File. College completion was represented by the percentage of	American Community Survey, 2015

	individuals living in the ZIP-code who completed at least a bachelor's degree.<br><br>For the difference-in-difference analyses, ZIP-code level college completion was re-coded into quartiles. At the ZIP-code level, 12,383 person-months (0.07%) were missing an associated college completion rate. These were excluded from the analyses.	
County-level COVID-19 Death Rates	Identified from publicly available data from the New York Times, who tracked and published monthly COVID-19 death rates throughout the duration of the pandemic at the county level. County-level death rates were cross-referenced to the county of residence associated with each individual from that year's Medicare Enrollment File. Data from years before 2020 were coded as 0 for this covariate.<br><br>For the difference-in-difference analyses, any missing data was recoded to 0.	New York Times Data Release, 2020-2022

eAppendix

Difference-in-Difference Calculations

Difference-in-difference study designs were employed in this analysis to compare changes in outcome measures among patients treated in dialysis facilities located in hospital referral regions (HRRs) selected for ETC model participation versus those treated at facilities located in control regions. We selected this analytic approach due to analyses of pre-model trends, which demonstrated baseline differences in the rate of home dialysis utilization across ETC and control regions, as well as significant differences in the patient population composition. Our approach assesses the changes in outcomes in ETC and control regions after random assignment. The primary difference-in-difference equation employed for individual *i*, in census region *j*, in month *t*:

$$Outcome_{ijt} = \beta_0 + \beta_1 ETC_j + \beta_2 Post_t + \beta_3 (ETC_j \times Post_t) + \beta_4 X_{ijt} + \delta_j + \eta_t + \varepsilon_{ijt} \quad (1)$$

where *ETC* designates whether the patient engaged with dialysis in an ETC region, $Post_t$ is an indicator for whether month *t* is after implementation of the ETC model (January 2021). The coefficient β_3 is the main parameter of interest and measures the effect of the ETC model after its implementation. X_{ijt} are sociodemographic and health status covariates (including age, sex, race and ethnicity, dual Medicare/Medicaid enrollment, reason for Medicare entitlement, ZIP-code level poverty, college completion, and monthly COVID-19 death rates). δ_j and η_t are census region and monthly fixed effects, respectively. We used heteroskedastic robust standard errors clustered at the HRR to account for potential serial correlation within regions over time. We employ census region fixed effects to account for the level of model randomization (HRRs were randomly selected within census region) while preserving the ability to observe across-HRR differences in outcomes. Standard errors are clustered at the level of treatment randomization, the HRR. We employed the "reghdfe" command for fitting the model in Stata.

Triple differences models include an additional interaction of ETC and $Post_t$ indicators on sociodemographic characteristics of interest, including age (<65 / >=65), sex, race/ethnicity (non-

© 2024 Koukounas KG et al. *JAMA Health Forum.*

Hispanic Black, White, Hispanic), dual status (non-dual, any dual, partial dual, full dual) and poverty quartile. This equation takes the form of:

$$Outcome_{ijt} = \beta_0 + \beta_1 ETC_j + \beta_2 Post_t + \beta_3 (ETC_j \times Post_t) + \beta_4 Race_i + \beta_5 (ETC_j \times Race_i) + \beta_6 (Post_t \times Race_i) + \beta_7 (ETC_j \times Post_t \times Race_i) + \beta_4 X_{ijt} + \delta_j + \eta_t + \varepsilon_{ijt} \quad (2)$$

where $Race_i$ is an example indicator for one of the sociodemographic categories of interest, race/ethnicity (indicators for non-Hispanic Black race and Hispanic ethnicity). The coefficient of the triple difference, β_7, will determine whether the ETC model led to significant changes in the magnitude of disparity between White and Black or White and Hispanic patients. All other covariates align in definition with equation 1 above.

Extended Results & Sensitivity Analyses

We conducted a series of sensitivity analyses to test the robustness of our results. We first generated our paper's Table 1, limiting to the active-model time period (2021-2022, **eTable 3**) and pre-policy (2017-2020, **eTable 4**), to evaluate whether patient characteristic composition significantly changed. We do not observe any significant adjustments in the composition of the patient population before versus during the model.

eTable 3. Individual-Level Distribution of Characteristics Across ETC and Non-ETC Assigned Regions, Active-Model Time Period (2021-2022)

	Non-ETC Region	ETC Region
Individuals, No. (%)	252,378 (66.3)	128,360 (33.7)
Age, Mean (SD)	62.9 (14.4)	62.7 (14.4)
Female, No. (%)	105,930 (42.0)	54,342 (42.3)
Race / Ethnicity		
Asian	11,863 (4.7)	4,355 (3.4)
Hispanic	23,540 (9.3)	8,125 (6.3)
Native American	2,890 (1.1)	3,318 (2.6)
Non-Hispanic Black	76,553 (30.3)	45,755 (35.6)
Other	7,322 (2.9)	2,911 (2.3)
Unknown	7,381 (2.9)	3,766 (2.9)
White	122,829 (48.7)	60,130 (46.8)
Region		
Midwest	51,241 (20.3)	22,181 (17.3)
North	37,132 (14.7)	22,234 (17.3)
South	109,440 (43.4)	61,195 (47.7)
West	54,565 (21.6)	22,750 (17.7)
Medicare Entitlement Reason		
ESRD	246,883 (97.8)	125,836 (98.0)
Aged	126,801 (50.2)	64,002 (49.9)
Disabled	74,611 (29.6)	38,448 (30.0)
Social Determinants of Health Factors		
ZIP-Code Level Poverty	17.7 (9.8)	17.7 (9.9)
ZIP-Code Level College Completion	22.8 (13.3)	22.8 (12.5)
Dual (Any)	115,527 (45.8)	56,382 (43.9)

Dual (Partial)	19,630 (7.8)	11,757 (9.2)
Dual (Full)	95,897 (38.0)	44,625 (34.8)

eTable 4. Individual-Level Distribution of Characteristics Across ETC and Non-ETC Assigned Regions, Pre-ETC Model Time Period (2017 - 2020)

	Non-ETC Region	ETC Region
Individuals, No. (%)	398,322 (66.9)	198,127 (33.2)
Age, Mean (SD)	62.0 (14.4)	61.8 (14.4)
Female, No. (%)	168,192 (42.2)	84,036 (42.4)
Race / Ethnicity		
Asian	17,132 (4.3)	6,063 (3.1)
Hispanic	34,469 (8.7)	11,814 (6.0)
Native American	4,633 (1.2)	4,957 (2.5)
Non-Hispanic Black	125,907 (31.6)	72,532 (36.6)
Other	10,457 (2.6)	4,108 (2.1)
Unknown	8,064 (2.0)	4,009 (2.0)
White	197,660 (49.6)	94,644 (47.8)
Region		
Midwest	82,871 (20.8)	35,321 (17.8)
North	58,177 (14.6)	33,936 (17.1)
South	175,461 (44.1)	95,032 (48.0)
West	81,813 (20.5)	33,838 (17.1)
Medicare Entitlement Reason		
ESRD	384,174 (96.4)	191,938 (96.9)
Aged	188,041 (47.2)	92,959 (46.9)
Disabled	131,561 (33.0)	66,169 (33.4)
Social Determinants of Health Factors		
ZIP-Code Level Poverty	18.2 (9.9)	18.3 (10.2)
ZIP-Code Level College Completion	22.6 (13.5)	22.4 (12.5)
Dual (Any)	173,110 (43.5)	83,859 (42.3)
Dual (Partial)	33,599 (8.4)	19,264 (9.7)
Dual (Full)	139,511 (35.0)	64,595 (32.6)

We conducted an event-study analysis to test for parallel pre-trends, a core assumption of the difference-in-difference framework. Event study analyses were constructed by interacting our ETC region indicator with a vector of quarter indicators for the study time period, as shown in the graphs in **eFigure 3** below. For both Home Dialysis and Kidney Transplant, pre-period trends are not significantly different from 0, as shown by the confidence intervals for each quarter-level estimate prior to 2021, providing us with confidence in the parallel nature of our pre-trends.

© 2024 Koukounas KG et al. *JAMA Health Forum.*

eFigure 3. Event Study Plots

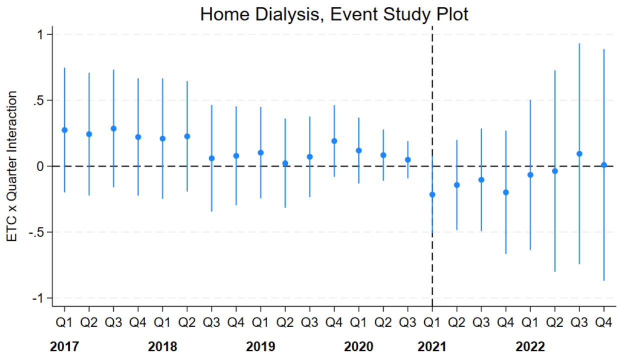

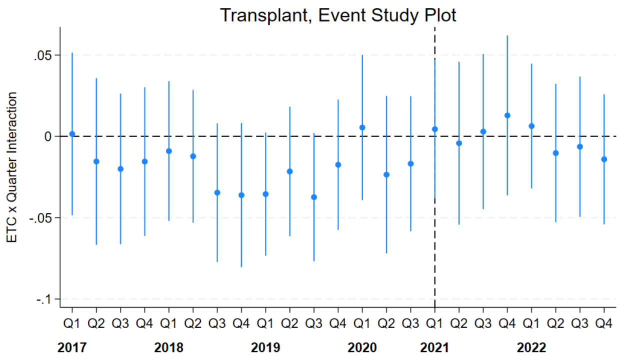

Expanded Triple Differences Results

eTable 5. Triple Difference-in-Difference Results for Home Dialysis Utilization (%) Among Kidney Failure Patients, Stratified by Social Determinants of Health Factors (2017-2022)

	ETC Regions			Control Regions			Difference-in-Difference	
	Pre-Policy	*Post-Policy*	*Diff. (p.p.)*	*Pre-Policy*	*Post-Policy*	*Diff. (p.p.)*	*Unadjusted*	*Adjusted*
Person-Months (N)	4,425,700	1,702,832		8,682,187	3,307,471			
Home Dialysis Utilization (%)								
**Age**								
<65	13.41	15.59	2.18	14.29	16.26	1.96	*Ref*	*Ref*
>=65	10.47	13.12	2.65	11.19	13.94	2.75	-0.32 [-0.93, 0.29]	-0.16 [-0.77, 0.44]
**Sex**								
Male	11.75	14.01	2.26	12.59	14.83	2.25	*Ref*	*Ref*
Female	12.50	14.72	2.22	13.26	15.35	2.09	0.11 [-0.39, 0.62]	0.05 [-0.45, 0.55]
**Race/Ethnicity**								
White	15.50	17.68	2.18	15.92	17.92	2.00	*Ref*	*Ref*

© 2024 Koukounas KG et al. *JAMA Health Forum.*

Non-Hispanic Black	8.62	10.23	1.62	9.24	10.98	1.74	-0.32 [-1.05, 0.41]	-0.25 [-1.05, 0.55]
Hispanic	9.96	11.5	1.55	10.51	11.67	1.16	0.14 [-0.78, 1.06]	-0.12 [-1.06, 0.83]
**Dual Status**								
Non-Dual (Any)	14.71	17.10	2.39	16.06	18.57	2.51	*Ref*	*Ref*
Dual (Any)	9.01	10.88	1.87	9.38	11.05	1.68	0.35 [-0.30, 1.00]	0.23 [-0.38, 0.83]
Partial Dual	9.79	11.28	1.49	10.19	11.80	1.61	-0.21 [-1.17, 0.76]	0.03 [-0.96, 1.02]
Full Dual	8.69	10.77	2.08	9.11	10.89	1.78	0.51 [-0.19, 1.21]	0.38 [-0.27, 1.03]
**ZIP-Code Level Poverty Quartile**								
Other Quartiles Poverty	12.66	14.81	2.15	13.31	15.58	2.27	*Ref*	*Ref*
Highest Quartile Poverty	10.41	12.67	2.26	11.60	13.26	1.65	0.73 [-0.50, 1.96]	0.44 [-0.79, 1.66]

© 2024 Koukounas KG et al. *JAMA Health Forum.*

eFigure 4. Unadjusted Home Dialysis Trends by Quarter, Stratified Analyses

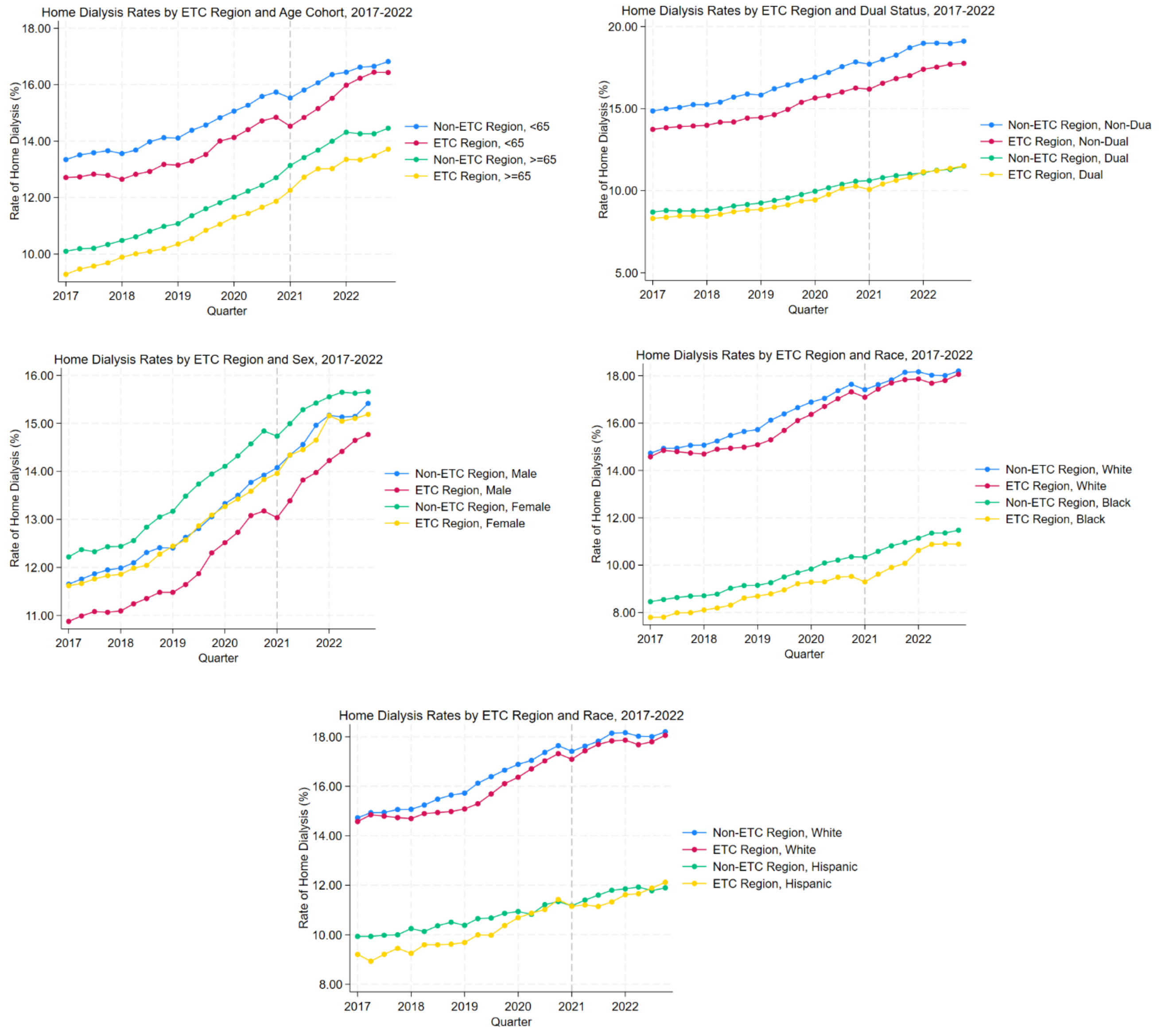

© 2024 Koukounas KG et al. *JAMA Health Forum.*

Medicare Switchers vs Stayers Expanded Analyses

To understand the implications of the expansion of MA to the kidney failure population on January 1, 2021 under the 21st Century Cures Act, we conducted a separate analysis that was restricted to beneficiaries with 12 months of continuous enrollment in traditional Medicare in the year 2020 (N=190,155) and evaluated selected one-year pre-policy (2020) and post-policy (2021) outcomes. These beneficiaries were classified into those that disenrolled to MA on Jan. 1st, 2021 ("switchers") and those that stayed in traditional Medicare ("stayers"), along with ETC and control region designations. Comparisons were made across demographic characteristics, as well as primary and secondary outcomes of interest. DiD analyses were not conducted on this population, as we could not track forward ETC region assignment and most study outcomes for MA switchers. We evaluate kidney transplant and mortality rates in the 2021 post-policy period for both cohorts, assuming ETC region designation remains constant from the last-observed dialysis location in December 2020.

eTable 6. Individual-Level Distribution of Characteristics Across ETC and Non-ETC Assigned Regions, Distributed by Staying in Traditional Medicare vs Switching to Medicare Advantage in Jan. 2021 (2020)

	ETC Regions		Control Regions	
	Stayers	*Switchers*	*Stayers*	*Switchers*
Individuals, No.	56,793	8,079	110,513	14,727
Age, Mean	61.17	58.19	61.48	57.80
Female, (%)	42.73	43.20	42.34	41.43
Race / Ethnicity (%)				
Asian	3.59	2.76	5.11	2.92
Hispanic	6.95	7.72	10.38	9.60
Native American	3.18	1.81	1.29	0.81
Non-Hispanic Black	40.10	57.82	33.74	53.24
Other	2.21	1.49	2.80	1.97
White	41.69	26.77	44.41	29.76
Unknown	2.28	1.63	2.27	1.70
Region (%)				
Midwest	16.18	12.39	19.14	12.65
North	17.01	9.12	13.97	7.52
South	48.75	64.31	44.25	68.52
West	18.06	14.17	22.64	11.31
Medicare Entitlement Reason (%)				
ESRD	99.42	99.65	99.32	99.47

© 2024 Koukounas KG et al. *JAMA Health Forum.*

Aged	44.86	30.07	45.55	27.96
Disabled	41.31	58.00	40.88	59.29
Social Determinants of Health Factors (%)				
ZIP-Code Level Poverty	18.39	20.79	18.18	19.91
ZIP-Code Level College Completion	22.33	20.62	22.54	20.84
Dual (Any)	46.09	53.46	47.43	53.11
Dual (Partial)	13.09	23.46	11.28	23.22
Dual (Full)	33.00	30.00	36.15	29.90

eTable 7. Pre-Period (2020) Person-Month Level Difference in Outcomes Between those who Stayed in Traditional Medicare vs those who Switched to Medicare Advantage in Jan. 2021, (2020)

	ETC Regions			Control Regions		
	Stayers	*Switchers*	*Difference*	*Stayers*	*Switchers*	*Difference*
Person-Months	682,550	97,024		1,325,244	176,684	
Home Dialysis (%)	13.39	10.68	2.71	14.27	11.16	3.11
Hospital Admissions (per 1000)	80.56	83.80	-3.24	81.51	81.92	-0.41

For the same population studied above (those that have 12 months of continuous enrollment in 2020), we then looked forward for one year (through 2021) to track differences in outcomes among those who stayed in traditional Medicare ("stayers") compared to those who switched to Medicare Advantage on Jan. 1st, 2021 ("switchers"). Due to data availability constraints, we were only able to study the outcomes of Kidney Transplant and 3-month mortality for these two populations, with results shown below.

© 2024 Koukounas KG et al. *JAMA Health Forum.*

eTable 8. Post-Period (2021) Person-Month Level Difference in Outcomes Between those who Stayed in Traditional Medicare vs those who Switched to Medicare Advantage in Jan. 2021, (2021)

	ETC Regions			Control Regions		
	Stayers	*Switchers*	*Difference*	*Stayers*	*Switchers*	*Difference*
Person-Months	604,428	88,306		1,168,270	160,907	
Kidney Transplant (%)	0.47	0.40	0.07	0.45	0.39	0.06
3-Month Mortality (%)	4.75	4.19	0.55	4.88	4.08	0.80

© 2024 Koukounas KG et al. *JAMA Health Forum.*

Incident Patient Analyses

We then limited our study population to a proxy for incident kidney failure patients, defined as the first month in the Medicare enrollment file where individuals demonstrated Medicare eligibility due to diagnosis of ESRD. For these patients, we pulled the first three months following incidence and evaluated our study outcomes, to align with previously published research that limits to incident kidney failure patients. We observe limited difference in the characteristics of the incident kidney failure population in comparison to our broader cohort (**eTable 9**). For outcomes, the majority of our results remain non-significant when limited to the incident population, however we do observe unadjusted home dialysis use results in line with Johanson et. al.'s published estimates[7] (**eTable 10**).

eTable 9. Individual-Level Distribution of Characteristics Across ETC and Non-ETC Assigned Regions, Among Incident Kidney Failure Patients (2017-2022)[1]

	Non-ETC Region	ETC Region
Individuals, No. (%)	162,076 (66.8)	80,686 (33.2)
Age, Mean (SD)	63.1 (14.6)	62.9 (14.6)
Female, No. (%)	66,452 (41.0)	33,588 (41.6)
Race / Ethnicity		
Asian	6,734 (4.2)	2,451 (3.0)
Hispanic	13,953 (8.6)	4,754 (5.9)
Native American	1,923 (1.2)	1,947 (2.4)
Non-Hispanic Black	39,938 (24.6)	23,775 (29.5)
Other	4,449 (2.7)	1,789 (2.2)
White	90,705 (56.0)	43,790 (54.3)
Unknown	4,374 (2.7)	2,180 (2.7)
Region		
Midwest	34,646 (21.4)	14,664 (18.2)
North	22,083 (13.6)	13,368 (16.6)
South	71,428 (44.1)	38,193 (47.3)
West	33,919 (20.9)	14,461 (17.9)
Medicare Entitlement Reason		
ESRD	161,784 (99.8)	80,573 (99.9)
Aged	85,958 (53.0)	42,696 (52.9)
Disabled	36,736 (22.7)	18,540 (23.0)
Social Determinants of Health Factors		
ZIP-Code Level Poverty	17.7 (9.7)	17.7 (9.9)

© 2024 Koukounas KG et al. *JAMA Health Forum.*

ZIP-Code Level College Completion	22.5 (13.2)	22.4 (12.2)
Dual (Any)	63,494 (39.2)	30,938 (38.3)
Dual (Partial)	8,885 (5.5)	5,282 (6.5)
Dual (Full)	54,609 (33.7)	25,656 (31.8)

[1] Patients who were incident in the last 3 months of 2020 and 2022 were excluded from the analysis to address data censoring concerns.

eTable 10. Difference-in-Difference Results Among Incident Kidney Failure Patients, Months 1-3 Post Treatment Incidence (2017-2022) [1]

	ETC Regions			Control Regions			Difference-in-Difference	
	Pre-Policy	*Post-Policy*	*Diff. (p.p.)*	*Pre-Policy*	*Post-Policy*	*Diff. (p.p.)*	*Unadjusted*	*Adjusted*
Person-Months (N)	215,534	82,671		431,113	165,891			
Home Dialysis (%)	14.11	16.40	2.29	15.23	16.35	1.12	1.16 [0.11, 2.21]	0.82 [-0.27, 1.90]
Kidney Transplant (%)	0.15	0.10	-0.05	0.14	0.09	-0.05	0.003 [-0.03, 0.04]	0.003 [-0.03, 0.04]
3-Month Mortality (%)	2.40	4.15	1.75	2.40	4.48	2.08	-0.33 [-0.64, -0.02]	-0.34 [-0.64, -0.03]
Hospital Admissions (per 1000)	178.25	176.59	-1.66	177.61	184.16	6.55	-8.37 [-14.18, 2.56]	-6.96 [-13.00, -0.93]
Disenrollment to MA (%)	0.39	2.03	1.64	0.53	2.21	1.68	-0.05 [-0.44, 0.35]	-0.03 [-0.41, 0.36]

[1] Patients who were incident in the last 3 months of 2020 and 2022 were excluded from the analysis to address data censoring concerns.

Sensitivity Analyses

We conducted a sensitivity analyses for policy implementation date by changing the pre-period to active period cutoff point. In the main analysis, we use January 2021, the date the ETC model launched and began outcome measurement, to separate the pre-period from the ETC-period. For our sensitivity analyses, we changed this date to be July 2019 (**eTable 11**), the date the ETC model was announced by CMS, and September 2020 (**eTable 12**), the date the randomized HRRs were announced for model participation. The results observed from these two analyses were not statistically significant, and did not meaningfully differ from what was observed in the primary work.

© 2024 Koukounas KG et al. *JAMA Health Forum.*

eTable 11. Difference-in-Difference Results Among Kidney Failure Patients, Sensitivity Analysis Using July 2019 Model Announcement Date (2017-2022)

	ETC Regions			Control Regions			Difference-in-Difference	
	Pre-Policy	*Post-Policy*	*Diff. (p.p.)*	*Pre-Policy*	*Post-Policy*	*Diff. (p.p.)*	*Unadjusted*	*Adjusted*
Person-Months (N)	2,771,926	3,356,628		5,466,636	6,523,022			
Home Dialysis (%)	11.56	13.63	2.06	12.35	14.41	2.06	-0.01 [-0.38, 0.37]	0.11 [-0.26, 0.49]
Kidney Transplant (%)	0.28	0.31	0.04	0.28	0.31	0.03	-0.01 [-0.03, 0.01]	-0.01 [-0.03, 0.01]
3-Month Mortality (%)[1]	2.63	3.51	0.88	2.71	3.61	0.90	0.02 [-0.05, 0.09]	0.01 [-0.06, 0.08]
Hospital Admissions (per 1000)	119.20	117.64	-1.56	121.87	120.79	-1.08	0.54 [-1.64, 2.72]	-0.56 [-3.02, 1.91]
Disenrollment to MA (%)	0.16	0.90	0.74	0.25	0.94	0.69	-0.05 [-0.18, 0.08]	-0.05 [-0.18, 0.07]

[1] For the 3-month mortality outcome, data from the last 3 months of 2022 (October, November, December) was removed to account for data censoring, given that we lacked 2023 data on date of death, and thus were likely undercounting the 3-month rate of death in these last 3 months.

eTable 12. Difference-in-Difference Results Among Kidney Failure Patients, Sensitivity Analysis Using September 2020 HRR Randomization Announcement Date (2017-2022)

	ETC Regions			Control Regions			Difference-in-Difference	
	Pre-Policy	*Post-Policy*	*Diff. (p.p.)*	*Pre-Policy*	*Post-Policy*	*Diff. (p.p.)*	*Unadjusted*	*Adjusted*
Person-Months (N)	4,064,623	2,063,909		7,982,996	4,006,662			
Home Dialysis (%)	11.95	14.16	2.21	12.75	14.92	2.17	-0.05 [-0.49, 0.39]	0.19 [-0.28, 0.67]
Kidney Transplant (%)	0.29	0.32	0.03	0.29	0.30	0.01	-0.02 [-0.04, 0.01]	-0.02 [-0.04, 0.01]

© 2024 Koukounas KG et al. *JAMA Health Forum.*

3-Month Mortality (%)[1]	2.73	3.92	1.19	2.81	4.04	1.23	0.04 [-0.05, 0.13]	0.06 [-0.03, 0.15]
Hospital Admissions (per 1000)	117.25	120.50	3.25	119.97	123.90	3.93	0.83 [-1.51, 3.17]	-0.56 [-3.17, 2.04]
Disenrollment to MA (%)	0.16	1.37	1.21	0.24	1.40	1.16	-0.06 [-0.27, 0.16]	-0.01 [-0.21, 0.19]

[1] For the 3-month mortality outcome, data from the last 3 months of 2022 (October, November, December) was removed to account for data censoring, given that we lacked 2023 data on date of death, and thus were likely undercounting the 3-month rate of death in these last 3 months.

Finally, we tested our difference-in-difference model specification using HRR, rather than census region, fixed effects, resulting in the analysis of within-HRR differences in outcomes across the pre-period and active-model time periods. The results remain non-significant, lending credence to our null finding regarding the ETC model's impact.

eTable 13. Difference-in-Difference Results Among Kidney Failure Patients using HRR fixed effects (2017-2022)

	ETC Regions			Control Regions			Difference-in-Difference	
	Pre-Policy	*Post-Policy*	*Diff. (p.p.)*	*Pre-Policy*	*Post-Policy*	*Diff. (p.p.)*	*Unadjusted*	*Adjusted*
Person-Months (N)	4,425,700	1,702,832		8,682,187	3,307,471			
Home Dialysis (%)	12.07	14.31	2.24	12.87	15.05	2.18	0.13 [-0.33, 0.59]	0.02 [-0.45, 0.48]
Kidney Transplant (%)	0.29	0.32	0.03	0.29	0.30	0.01	0.01 [-0.01, 0.04]	0.01 [-0.01, 0.04]
3-Month Mortality (%)	2.82	3.94	1.12	2.90	4.04	1.14	-0.02 [-0.12, 0.09]	-0.03 [-0.12, 0.07]
Hospital Admissions (per 1000)	117.19	121.34	4.15	119.85	125.04	5.19	-1.29 [-3.80, 1.22]	0.93 [-3.39, 1.54]
Disenrollment to MA (%)	0.39	1.04	0.65	0.45	1.08	0.64	0.02 [-0.10, 0.13]	0.004 [-0.10, 0.11]

© 2024 Koukounas KG et al. *JAMA Health Forum.*

[1] For the 3-month mortality outcome, data from the last 3 months of 2022 (October, November, December) was removed to account for data censoring, given that we lacked 2023 data on date of death, and thus were likely undercounting the 3-month rate of death in these last 3 months.

© 2024 Koukounas KG et al. *JAMA Health Forum.*

eReferences

1. Centers for Medicare & Medicaid Services. ESRD treatment choices (ETC) Model: CMS Innovation Center. Accessed October 12, 2023. https://innovation.cms.gov/innovation-models/esrd-treatment-choices-model

2. Centers for Medicare & Medicaid Services. Final Rule: Medicare Program; End-Stage Renal Disease Prospective Payment System, Payment for Renal Dialysis Services Furnished to Individuals with Acute Kidney Injury, End-Stage Renal Disease Quality Incentive Program, and End-Stage Renal Disease Treatment Choices Model. Published online 2021. Accessed October 12, 2023. https://www.federalregister.gov/documents/2022/11/07/2022-23778/medicare-program-end-stage-renal-disease-prospective-payment-system-payment-for-renal-dialysis

3. Centers for Medicare & Medicaid Services. End-Stage Renal Disease Treatment Choices (ETC) Model, Performance Payment Adjustment (PPA) Report User Guide (Measurement Years 1-2). Published online 2022. Accessed October 12, 2023. https://www.cms.gov/priorities/innovation/media/document/etc-4i-ppa-report-user-guide-my1-2

4. eCFR :: 42 CFR Part 512 Subpart C -- ESRD Treatment Choices Model. Accessed March 26, 2024. https://www.ecfr.gov/current/title-42/chapter-IV/subchapter-H/part-512/subpart-C

5. Centers for Medicare & Medicaid Services. Medicare Claims Processing Manual; Chapter 8 - Outpatient ESRD Hospital, Independent Facility, and Physician/Supplier Claims. Published online February 22, 2024. Accessed March 26, 2024. https://www.cms.gov/regulations-and-guidance/guidance/manuals/downloads/clm104c08.pdf

6. Centers for Medicare & Medicaid Services. *End-Stage Renal Disease Treatment Choices (ETC) Model, Performance Payment Adjustment (PPA) Report User Guide (Measurement Years 1-2).* CMS Innovation Center; 2022. Accessed March 2, 2024. https://www.cms.gov/priorities/innovation/media/document/etc-4i-ppa-report-user-guide-my1-2

7. Johansen KL, Li S, Liu J, et al. Association of the End-Stage Renal Disease Treatment Choices Payment Model With Home Dialysis Use at Kidney Failure Onset From 2016 to 2022. *JAMA Network Open*. 2023;6(2):e230806. doi:10.1001/jamanetworkopen.2023.0806

© 2024 Koukounas KG et al. *JAMA Health Forum.*
