# Supplementary material for: Pay-for-Performance Incentives for Home Dialysis Use and Kidney Transplant
Source: JAMA Health Forum. 2024 Jun 30;5(6.9):e242055. doi: 10.1001/jamahealthforum.2024.2055 (PMC11215557; doi:10.1001/jamahealthforum.2024.2055)
Supplement: Supplement 2. — Data Sharing Statement [file jamahealthforum-e242055-s002.pdf]

Data Sharing Statement

Koukounas. Pay-for-Performance Incentives for Home Dialysis Use and Kidney Transplant. *JAMA Health Forum*. Published June 30, 2024. doi:10.1001/jamahealthforum.2024.2055

Data
Data available: No

Additional Information
Explanation for why data not available: The data used for the study is claims data, and thus cannot be made public at the patient-level due to data sharing use agreements.
